# Supplementary material for: MSnLib: efficient generation of open multi-stage fragmentation mass spectral libraries
Source: Nat Methods. 2025 Sep 15;22(10):2028–31. doi: 10.1038/s41592-025-02813-0 (PMC12510872; doi:10.1038/s41592-025-02813-0)
Supplement: Supplementary file 1 — Supplementary Notes 1–8, Supplementary Figs. 1–15, Supplementary Tables 1 and 2 [file 41592_2025_2813_MOESM1_ESM.pdf]

nature methods

Supplementary information https://doi.org/10.1038/s41592-025-02813-0

MS^nLib: efficient generation of open multi-stage fragmentation mass spectral libraries

In the format provided by the authors and unedited

Table of Content

Supplementary Note 1: Compound libraries **3**

Supplementary Fig. 1. Insights into the chemical properties covered by the seven compound libraries ENAMDISC, MCEBIO, MCESCAF, ENAMMOL, NIHNP, MCEDRUG, and OTAVAPEP. **4**

Supplementary Note 2: Metadata cleanup & sequence generation **5**

Supplementary Fig. 2. Metadata Prefect flow screenshot server. **6**

Supplementary Note 3: Flow injection-data-dependent acquisition MSn method development **6**

Supplementary Fig. 3. Comparison of signal-to-noise ratios (SN) in MS3 spectra acquired with three methods differing by their fragmentation scan parameters (MS level > 2). **8**

Supplementary Fig. 4. Histograms of all MS3 scans in 22 samples show the impact of the removal of static noise signals 149.666-149.737 and 173.509-173.538 **9**

Supplementary Table 1. A summary of the MS1 instrument method including the dynamic exclusion. **10**

Supplementary Table 2. A summary of the MSn instrument method. **11**

Supplementary Note 4: Automatic MSn tree library generation and data evaluation in mzmine **12**

Supplementary Fig. 5. Feature table in mzmine. **14**

Supplementary Fig. 6. Quality check and merging within mzmine. **15**

Supplementary Note 5: MSnLib results **16**

Supplementary Fig. 7. Ratios of detected compounds in both ion modes for 384-well plates 1-3 of the MCEBIO. **16**

Supplementary Fig. 8. Ratios of detected compounds in both ion modes (both, only one, or missing) for 384-well plates 1-2 of the MCESCAF. **17**

Supplementary Fig. 9. Ratios of detected compounds in both ion modes (both, only one, or missing) for 384-well plate 1 of the OTAVAPEP. **18**

Supplementary Fig. 10. Ratios of detected compounds in both ion modes (both, only one, or missing) for 96-well plates 1-6 and 8-15 of the NIHNP. **19**

Supplementary Fig. 10 (continued). Ratios of detected compounds in both ion modes (both, only one, or missing) for 96-well plates 1-6 and 8-15 of the NIHNP. **20**

Supplementary Fig. 11. Ratios of detected compounds in both ion modes (both, only one, or missing) for 384-well plates 1-4 of the ENAMDISC. **21**

Supplementary Fig. 11 (continued). Ratios of detected compounds in both ion modes (both, only one, or missing) for 384-well plates 1-4 of the ENAMDISC. **22**

Supplementary Fig. 12. Ratios of detected compounds in both ion modes (both, only one, or missing) for 384-well plates 1-3 of the ENAMMOL. **23**

Supplementary Fig. 13. Ratios of detected compounds in both ion modes (both, only one, or missing) for 384-well plates 1-2 of the MCEDRUG. **24**

Supplementary Fig. 14. An example of an MSn tree for the peptide Val-Tyr-Val. **25**

Supplementary Fig. 15. The MSn tree visualizer in mzmine (screenshot). **25**

Supplementary Note 6: Quality check for the automatic spectral extraction **26**
Supplementary Note 7: Matching to a public metabolomics dataset **27**
Supplementary Note 8: Outlook **27**
References **29**

Supplementary Note 1: Compound libraries

For MS^nLib, 7 different compound libraries were analyzed. Out of 37,829 compounds, 34,413 are unique structures based on their InChIKey strings, taking stereoisomers into account. Accordingly, 2,250 structures are included in multiple compound libraries (up to 3 times), with the highest overlap between the MCEBIO and the MCEDRUG libraries with 2,038 structures in common. **Extended Data Table 1** lists more library metadata, for example, the catalog number, whether the respective compounds are registered as drugs (clinical phase 4) or in clinical trials (clinical phase 1-3), and whether they have entries in the LOTUS[1] database, linking known compound–species relationships. Accordingly, the seven compound libraries cover a chemical space of diverse compound classes with minimal overlap. More details about the mass distribution and functional groups can be found in **Extended Data Fig. 1 and Supplementary Fig. 1.** Most of the compounds are in the mass range of m/z 300–500 and have a LogP value between 0 and 5.

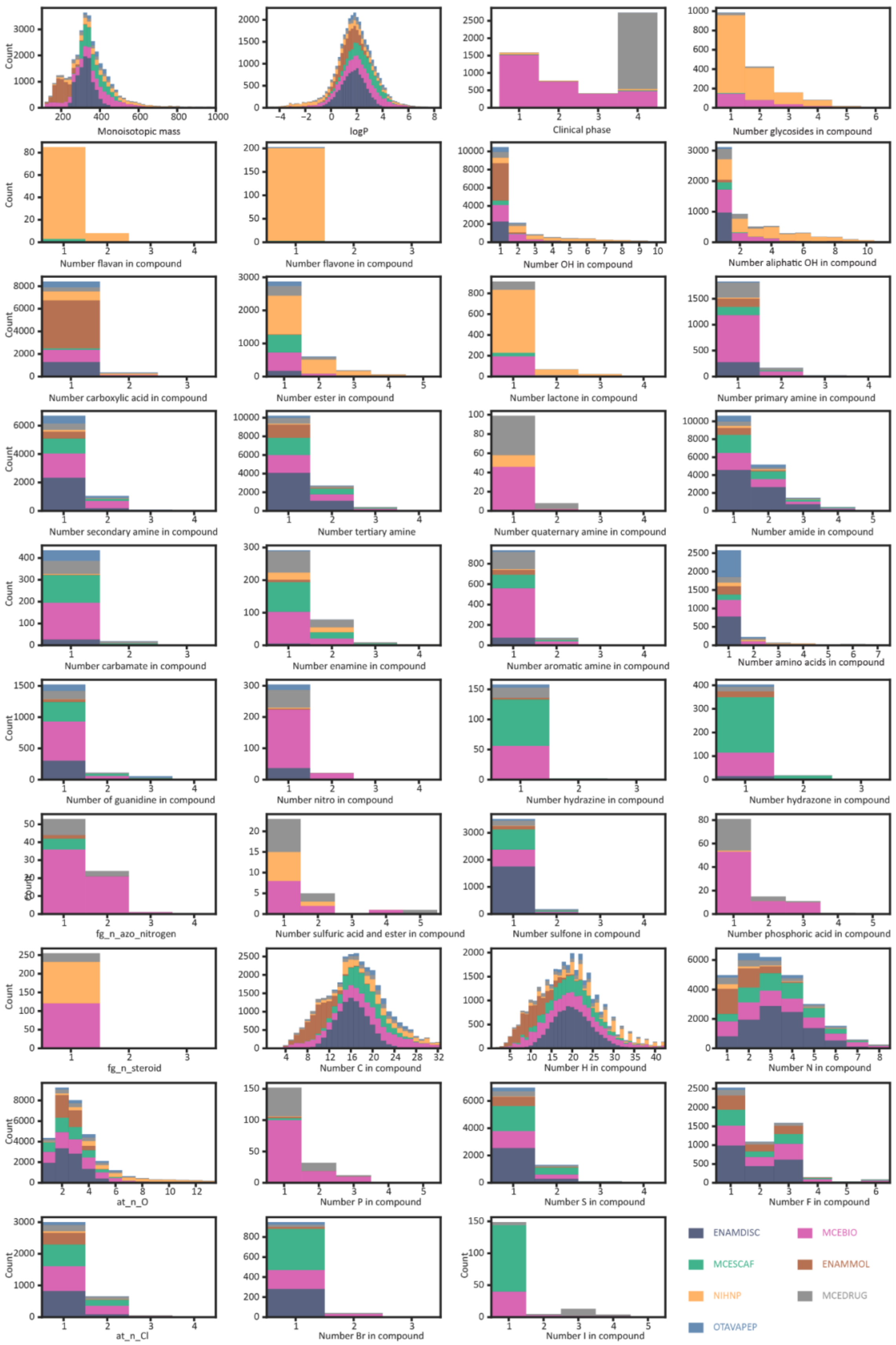

Supplementary Fig. 1. Insights into the chemical properties covered by the seven compound libraries ENAMDISC, MCEBIO, MCESCAF, ENAMMOL, NIHNP, MCEDRUG, and OTAVAPEP.
If a compound (same InChIKey) is included in multiple compound libraries, it is kept in the smallest one.

Supplementary Note 2: Metadata cleanup & sequence generation

The first crucial step during the cleanup of metadata is the standardization of chemical structures, often provided by sellers of compound libraries or accessed from public compound databases. The Python script presented here is based on the ChEMBL structure standardizer,[2] adding a step to remove salts from parent compounds. The majority of salts, such as tartrates, are most likely dissociated in solution before MS analysis and only later form ion adducts or clusters during ionization. The cleaned structure is then converted into canonical or isomeric SMILES, InChI, and InChIKey strings, and its exact monoisotopic neutral mass is calculated. This is important for the automatic library generation workflow, which annotates and extracts spectra based on their metadata. For the metadata, ion adducts and their expected m/z are irrelevant because they are computed in mzmine.

The metadata cleanup workflow also includes optional steps for enriching each compound with additional information and links found in compound databases and other web services, including PubChem,[3] ChEMBL,[4,5] UniChem,[6] DrugBank,[7] DrugCentral,[8] the Drug Repurposing Hub (Broad Institute, drug information),[9] LOTUS[1] on Wikidata, the Dictionary of Natural Products, the Natural Product Atlas,[10] and chemical classification by NPClassifier[11] and ClassyFire[12]. This search is based on unique identifiers related to the database or the first block of InChIKey (split InChIKey). The final table contains all input columns and new compound information, e.g., on a compound's use as a drug or its clinical phase and its potential biosynthetic origins. The whole workflow was implemented in Python 3.10, utilizing the Prefect library for task orchestration (**Supplementary Fig. 2**). In the event of a failing web service, the workflow can be applied iteratively to saved intermediate results. Upon a successful query, each service adds a data retrieval date, which can be used to flag data as stale and to rerun the corresponding service for compounds with outdated or missing metadata. Additional queries can be added or disabled easily thanks to pipeline's modular design. Required and optional metadata fields are described in the mzmine documentation (https://mzmine.github.io/mzmine_documentation/workflows/librarygeneration/library_generation.html). Briefly, the final format is a tab-separated (.tsv) or comma-separated (.csv) metadata sheet, with each row describing a relationship of a compound being contained in a sample. All compounds contained in the same mix point to the same unique sample ID, which should be a substring of each measurement, for example, sample_a1_id_pos.mzML and sample_a1_id_neg.mzML containing the unique sample ID '*sample_a1_id*'. This way the same metadata sheet can be used for different acquisition dates, methods, and instruments. The minimum required compound information for the subsequent automatic library generation is the neutral mass and a name. Structures such as SMILES, InChI, or at least the molecular formula are preferable and replace the neutral mass with its calculated value during the metadata cleanup.

Another Python script automatically generates data acquisition sequences based on the provided plate number, well location, and unique sample ID within the metadata sheet, as well as the autosampler location and instrument method files for both positive and negative ion modes. This script automatically groups entries by the unique sample ID, leading to one injection per sample and method (see **Fig. 1a** Acquisition sequence).

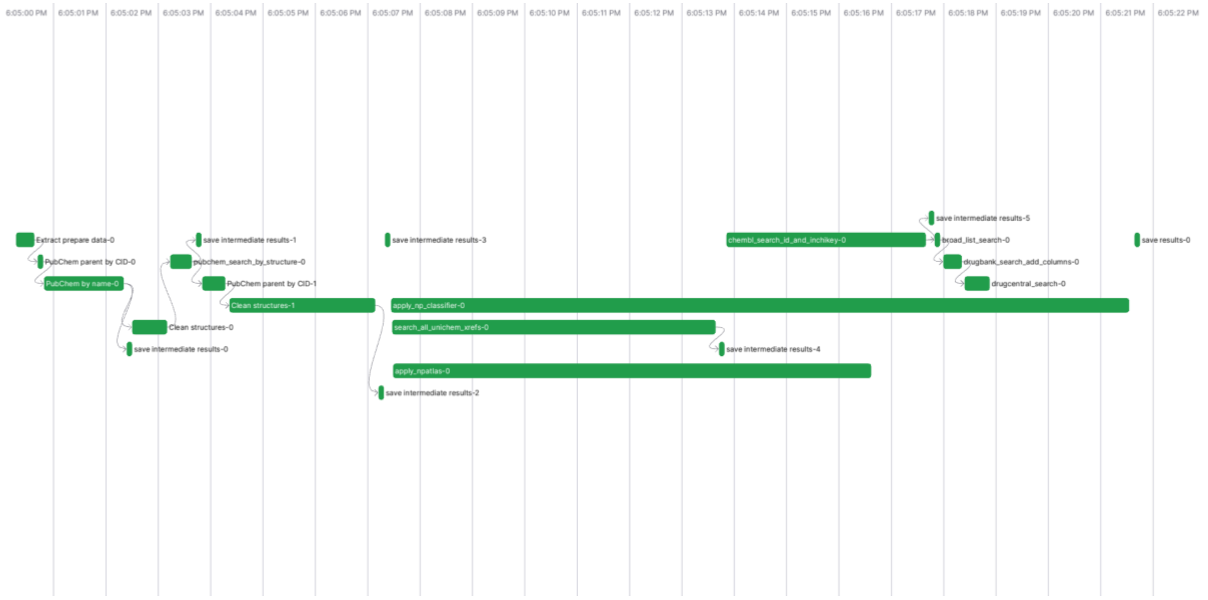

Supplementary Fig. 2. Metadata Prefect flow screenshot server.

The Prefect library is used for task orchestration, enabling the parallel query of various databases if the results are independent of each other. Otherwise, the task needs to be done first, which can be seen for the structure cleanup. Unichem is used to add unified identifiers before querying other databases. After finishing each task package, the intermediate results are saved.

Supplementary Note 3: Flow injection-data-dependent acquisition MS^n method development

We developed a dual-pump flow injection MS^n method to gather high-quality spectra on multiple adducts of up to ten compounds in a single injection. The acquisition of an ion's MS^n tree is time-consuming and can easily span multiple seconds, depending on the tree's depth and breadth. Additionally, the mass resolution and maximum injection time, together with the automatic gain control (AGC) setting, limit the scan rate to 5–8 Hz. For example, the presented method resulted in a maximum acquisition time of ~ 13 seconds per picked MS^2 precursor ion with up to MS^5, 25 precursor ions (5 for MS^3, 5×2 for MS^4, 10×1 MS^5), 75 spectra including three collision energies, and a 200 ms maximum injection time (schema shown in **Extended Data Fig. 2**. Consequently, this renders fast LC–MS unviable, considering its compound elution profiles in the lower seconds range. We optimized a high-throughput flow injection method to reach a rectangular-current intensity profile of 1.5 minutes within 3 minutes of total analysis time, including a washout phase (see **Fig. 1b**). The final method provided enough time for ten mixed compounds per injection, including multiple adducts, considering that only a few precursors trigger a full MS^n tree. This method is based on a dual-pump liquid chromatography system with a sample delivery pump and a secondary makeup pump to modulate the elution profile and improve washout behavior (see the Online Methods). Most crucially, the data-dependent acquisition (DDA) mode with combined static and dynamic precursor exclusion was used. The static exclusion list was generated for measured blanks (i.e. the dilution solvents) in each polarity. After that, mzmine was used to

extract all *m*/*z* values with a minimum intensity ten times lower than the intensity threshold set for the Orbitrap ID-X to trigger MS^2 and those detected in multiple blank injections. The dynamic exclusion was set to 1.2 min-width to guarantee better coverage of various ion adducts and compounds within the same run. This still allowed individual ions to be picked at the start and end of the elution peak.

A pilot study on 22 samples corresponding to 220 pure standards from the MCEBIO compound library was conducted to evaluate the influence of the AGC target and mass resolution, focusing on MS level ≥ 3. Accordingly, the settings for MS^1 and MS^2 remained unchanged whereas the other MS levels were affected. In Method 1, the AGC target was set to 60% (30k), which is higher than the recommendation for small molecules by the instrument's manufacturer, specifically MS3-OT AGC for small molecules between 20% and 40% (10–20k). In Method 2, the AGC target was increased to 100% (50k). In Method 3, the mass resolution was increased from 15k to 60k because the 128 ms scan time needed for 60k utilizes the 200 ms maximum injection time optimally. The comparison of all methods is summarized in **Extended Data Fig. 3a** by plotting the lowest-intensity signal, here defined as the noise level, of each MS^3 spectrum against its corresponding 1/injection time. A higher AGC target shifts the scan rate to longer injection times, resulting in lower noise levels. This can be explained by the internal processing in trap-based MS instruments, where measured intensities are divided by the injection time for response normalization. Similar trends toward even lower noise levels were achieved by increasing the mass resolution, which lowers the slope of the linear regression (Method 3, magenta). Overall, Method 3 resulted in higher signal-to-noise (SN) ratios, as exemplified in **Supplementary Fig. 3**, which compares all MS^3 spectra (several MS^3 precursors) of the same MS^2 precursor ion picked in all three methods. Here, Method 3 halved the noise level to 1E5, offering a better distinction between signals and noise. During data processing, mass detection in mzmine applied denoising with a threshold of 2.5 times the lowest signal in each scan to clean the spectra before exporting with our automatic library building workflow. A histogram in **Extended Data Fig. 3b** compares the number of signals in individual scans for all exported MS^{3-5} scans to measure spectral quality. Overall, the higher AGC and mass resolution yielded more spectra with more than ten fragment signals and increased the average number of MS^3 signals from 14 in Method 1 to 19 and 23 in Method 2 and 3, respectively.

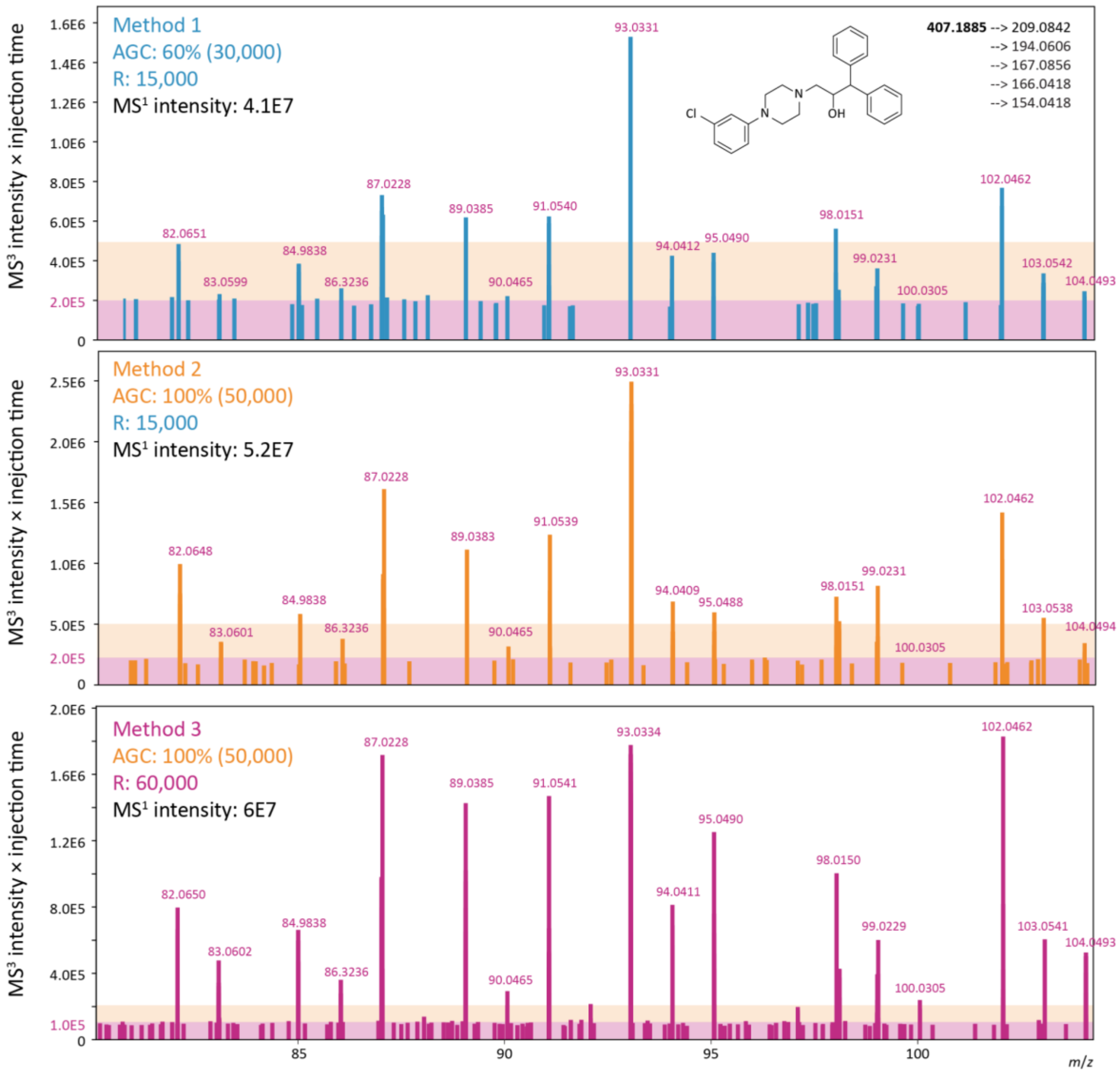

Supplementary Fig. 3. Comparison of signal-to-noise ratios (SN) in MS^3 spectra acquired with three methods differing by their fragmentation scan parameters (MS level > 2).

The MS^3 spectra of 5 MS^3 precursors for the compound ion m/z 407.1885 were acquired in three separate runs, always on the intensity plateau at similar MS^1 signal intensities. The m/z axis is zoomed to allow better differentiation of individual signals. A red box highlights the static noise level at the bottom of the spectra, here visual by the grassy noise signals. The yellow box highlights 2.5-times this noise level, i.e. the threshold used during mass detection in mzmine. Method 1 and 2 resulted in the same absolute noise level of 2E5, however, Method 2 boosted the signal intensities of all other signals by 80-100%. This doubling of the SN corresponds to the increased AGC from 60% to 100%. In Method 3, the higher mass resolution halved the noise level to 1E5 lifting previously lower abundant signals clearly above the applied noise level within mzmine.

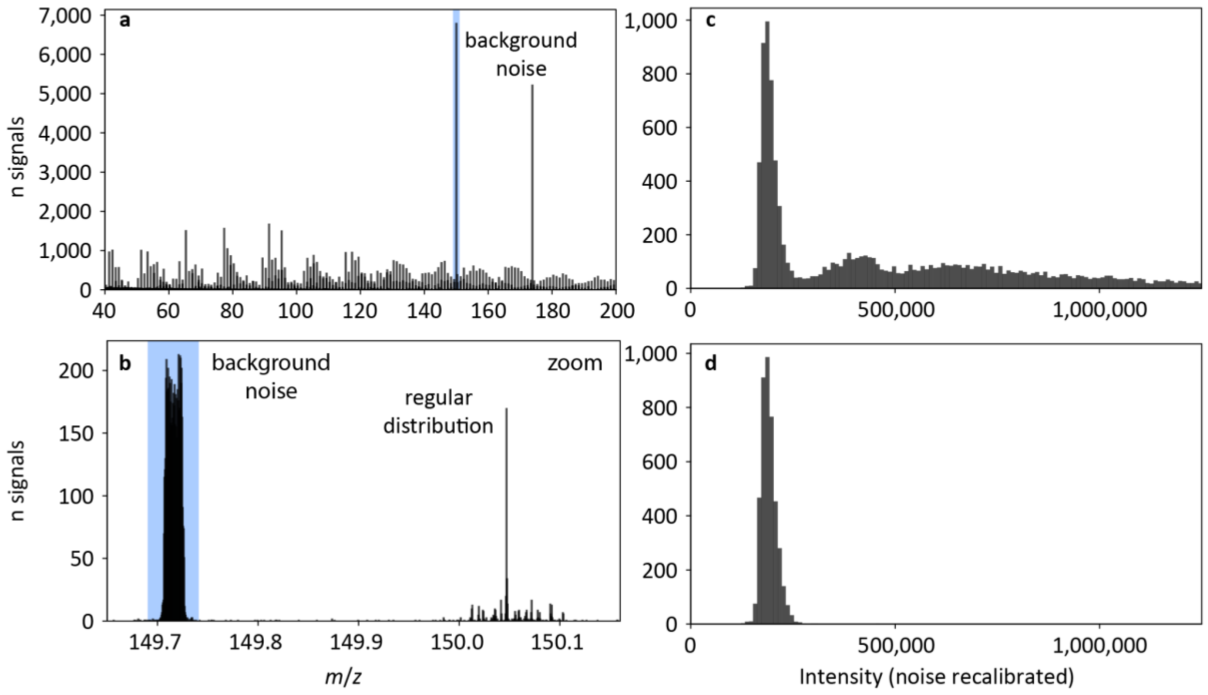

Supplementary Fig. 4. Histograms of all MS^3 scans in 22 samples show the impact of the removal of static noise signals 149.666-149.737 and 173.509-173.538

a, m/z histogram contains 2 major signals that were detected multiple times in all samples, m/z binning of 0.05. **b,** Zoomed-in version with finer binning of 0.0005, showing a broad signal distribution for the background noise in comparison to a regular signal distribution. **c,** The background signal intensity distribution for signals up to m/z 200 and a mass defect of 0.65 and 0.75 before filtering out the static noise signals. **d,** After signal removal, there is only one distribution that mostly constitutes spectral noise as only signals < m/z 200 with a mass defect between 0.65-0.75 were included. This range does not include many possible chemical formulas of singly-charged fragment ions. Considering MS level 3 there is a low chance for multiply-charged fragment ions.

Supplementary Table 1. A summary of the MS[1] instrument method including the dynamic exclusion.

Ion Source Properties	Setting
Ion Source Type	H-ESI
Positive ion (V)	3000
Negative ion (V)	2000
Sheath Gas (Arb)	25
Aus Gas (Arb)	5
Sweep Gas (Arb)	0
Ion Transfer Tube Temp (°C)	275
Vaporizer Temp (°C)	75
Internal Mass Calibration	Off or Start
**MS Scan Properties**	**MS1**
Detector Type	Orbitrap
Orbitrap Resolution	30,000
Use Quadrupole Isolation	✓
Scan Range (m/z)	115-2000
RF Lens (%)	50
AGC Target	4.00E+05
Maximum Injection Time (ms)	50
Microscans	1
Data Type	Profile
**Dynamic Exclusion Properties**	
Exclude after n Times	3
If occurs within (s)	200
Exclusion Duration (s)	70
Mass Tolerance (m/z)	0.2
**Isotope Exclusion (m/z window)**	2
**Targeted Mass Exclusion**	blank injection

Supplementary Table 2. A summary of the MS^n instrument method.

The MS^n level was set to a maximum MS^5. For each precursor, 3 different fragmentation experiments were conducted to cover 3 fragmentation energies. For MS^2, the assisted collision energy was used to get the best fragmentation scan for the following stages. Schema is shown in Extended Data Fig. 2.

	MS^n Stage			
**Data-Dependent MS^n Scan Properties**	**MS^2 (9 scans)**	**MS^3 (15 scans)**	**MS^4 (6 scans)**	**MS^5 (2 scans)**
Isolation Mode	Quadrupol	-	-	-
Isolation Window (m/z)	1.2	2	2.2	3
Isolation Offset	Off	Off	Off	Off
Activation Type	HCD	HCD	HCD	HCD
Collision Energy Mode Exp.1	Fixed	-	-	-
HCD Collision Energy Type Exp.1	Normalized	Normalized	Normalized	Normalized
HCD Collision Energies (%) Exp.1	20	20	20	40
Collision Energy Mode Exp.2	Assisted	-	-	-
HCD Collision Energy Type Exp.2	Normalized	Normalized	Normalized	Normalized
HCD Collision Energies (%) Exp.2	15, 30, 45, 60, 75	40	40	60
Collision Energy Mode Exp.3	Fixed	-	-	-
HCD Collision Energy Type Exp.3	Normalized	Normalized	Normalized	-
HCD Collision Energies (%) Exp.3	60	60	60	-
Detector Type	Orbitrap	Orbitrap	Orbitrap	Orbitrap
Orbitrap Resolution	15,000	60,000	60,000	60,000
First Mass Scan Range (m/z)	40	40	40	40
AGC Target	1.20E+04	5.00E+04	5.00E+04	5.00E+04
Maximum Injection Time (ms)<br>(negative)	50<br>(80)	200<br>(500)	200<br>(500)	200<br>(500)
Microscans	1	1	1	1
Data Type	Profile	Profile	Profile	
**Precursor Selection Range**				
Mass Range (m/z)	115-2000	90-2000	90-2000	150-2000
**Intensity Properties**				
Intensity Threshold<br>(negative)	600,000<br>(200,000)	20,000<br>(10,000)	20,000<br>(10,000)	20,000<br>(10,000)
**Targeted Mass Exclusion**		149.72, 173.52	149.72, 173.52	149.72, 173.52
**Precursor Ion Exclusion (m/z)**		8	8	8

Supplementary Note 4: Automatic MS^n tree library generation and data evaluation in mzmine

We implemented the automatic workflow for MS^n tree library generation into mzmine and applied it to all seven compound libraries and both ionization polarities. The new core additions to allow for the handling of multi-stage fragmentation data include the '*MS^n tree feature list builder*', a set of advanced algorithms for spectral merging on multiple MS levels for various fragmentation energies, and the export of MS^n tree libraries. Starting with the data import of converted open .mzML files or raw vendor formats, all spectra were denoised by removing signals below 2.5 times the lowest signal in each MS$^{1\text{-}n}$ scan (see **Supplementary Fig. 3**), accounting for the varying noise level due to ion trap-based normalization. This is illustrated in the **Extended Data Fig. 3a**, where the lowest signal intensity increases with shorter filling times due to the normalization processing of the vendor. This fact prevents the use of an absolute noise level. In less intense MS^n scans, broadly distributed noise signals were observed with an irregularly high mass defect for low m/z values (see **Supplementary Fig. 4**). The instrument vendor typically flags such artifacts in the spectra, but the ThermoRawFileParser failed to remove those artifacts in MS$^{3\text{-}n}$ during data conversion. Therefore, mzmine's '*Scan signal removal*' was applied to remove reoccurring known contaminant signals. Due to their uncommon mass defects, real spectral data should remain unaffected. This step should be used with caution, and only abundant frequently detected signals should be removed that cannot be explained by a subformula from the original structures. The '*MS^n tree feature list builder*' then extracted flow injection–MS^1 time traces for each MS^2 precursor and built MS^n ion trees. The resulting feature list was annotated by mzmine's '*Custom database search*' using the cleaned metadata sheet from the first step. **Supplementary Fig. 5** visualizes an example feature list with database annotation. Most crucially, the *unique_sample_id* column was selected to limit annotations to those samples that actually contain the corresponding compounds in the mix of 8 to 10 compounds. This step paired the monoisotopic mass from the metadata with a user-defined list of adducts to define the search space. Our advice is to search for a broad range of ion adducts, in-source fragments, and clusters in a first iteration before limiting the ion selection to those with the highest detection frequencies in the final library building workflow. Too many included adducts will yield error-prone spurious hits. Ion formation depends on the chemical structures analyzed, the solvents used, the sample matrix, and the ionization conditions (see the Online Methods). For MS^{n}Lib, we included six adduct types in positive ion mode as [M], $[M]^+$, $[M+H]^+$, $[M-H_2O]^+$, $[M-H_2O+H]^+$, $[M-2H_2O+H]^+$, $[M+NH_4]^+$, and $[M+Na]^+$ and five adducts in negative ion mode, namely [M], $[M]^-$, $[M-H]^-$, $[M+Cl]^-$, and $[M+FA]^-$. In mzmine, [M] refers to an ion that is intrinsically charged and might match compounds like quaternary amines. Subsequently, MS^n tree features annotated as specific ion species of the input compounds were exported to various open spectral library formats, e.g., .json (mzmine), .mgf, or .msp. This export took each annotated MS^n tree, scored and filtered the spectral quality of all fragmentation spectra (**Supplementary Fig. 6a,b**), and applied spectral merging on each MS level and fragmentation energy separately. The resulting list contains a merged and single best (highest total ion chromatogram (TIC) spectrum for each precursor ion and fragmentation energy, together with a merged spectrum across all fragmentation energies. Finally, a pseudo-MS^2 spectrum was created by merging the whole MS^n tree (**Supplementary Fig. 6c**). This spectrum can be useful as input to annotation tools that only support MS^2 data, such as SIRIUS[13] and GNPS Ion Identity Molecular Networking (IIMN)[14]. Before writing the new library entries, further quality checks were applied to the MS^1 and MS^n levels. First, precursor isolation purity was scored

within the corresponding MS^1 scans before and after each MS^n tree (**Supplementary Fig. 6a**). This percentage score takes a main signal in the center of the isolation window, divided by the intensity of all signals falling into the actual isolation window. Two options are provided to either skip or just flag library entries that exhibit low precursor purity. Our general approach was to flag such spectra and to split the library later to match the study requirements. Running the workflow in the mzmine batch mode applied all steps in a pipeline with reproducible results. This way, mzmine generated feature lists for intermediate steps facilitating the validation of results and backtracking of potential error sources (**Supplementary Fig. 5**). Finally, the workflow reimported the MS^n tree library as an automated parsing test. Positive and negative ionization modes were processed with separate mzmine batch configurations, due to the different adduct settings, resulting in individual libraries for each polarity. Each spectral library entry comprises metadata fields including the compound name, structural information, adduct type, collision energy, precursor m/z isolation window, quality check results, polarity, and spectrum type (single best scan or various merging types). Library-specific information can be added in the library export metadata, including the instrument type, contact details, and dataset ID, if data was uploaded to a public repository. The dataset ID also generates universal spectrum identifiers (USI) for each spectral library entry. USI facilitates traceability of FAIR data similar to a file path into the public domain, denoting the dataset, filename, and scan number.[15] Merged scans contain all USI of their source scans. The available metadata is useful to filter the MS^nLib into subsets, e.g., for specific instruments.

The processing time for 11,000 compounds in 1100 samples was approximately 8 minutes when performed on a consumer-grade laptop. More information is presented in the Online Methods. Being 480× faster than data acquisition allows for the scalable iterative optimization of the whole workflow.

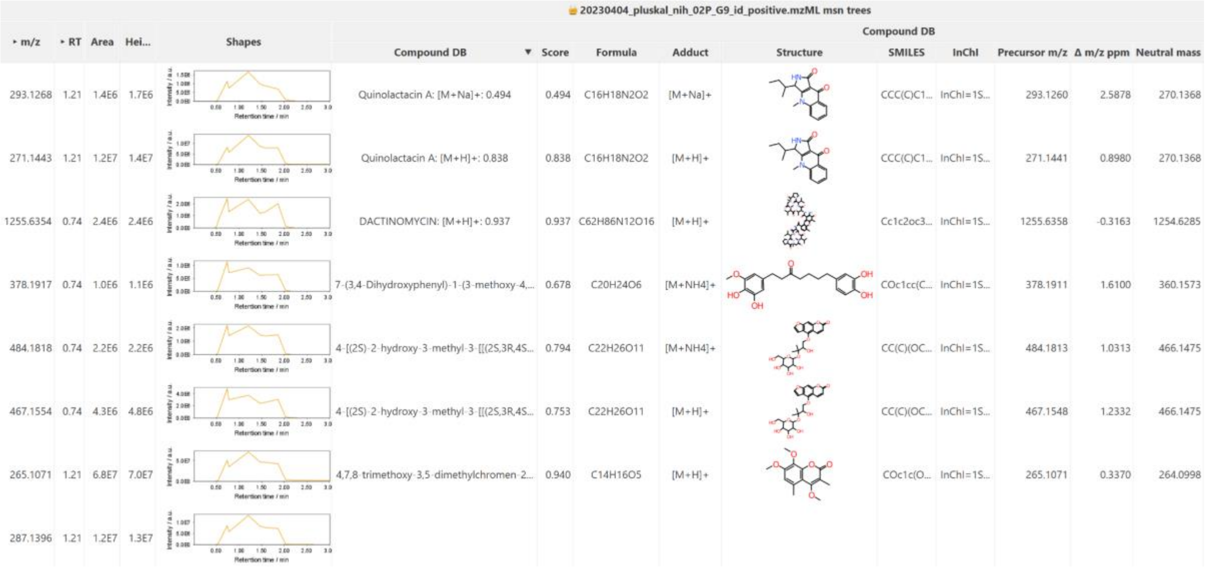

					20230404_pluskal_nih_02P_G9_id_positive.mzML msn trees									
					Compound DB									
m/z	RT	Area	Hei...	Shapes	Compound DB	Score	Formula	Adduct	Structure	SMILES	InChI	Precursor m/z	Δ m/z ppm	Neutral mass
293.1268	1.21	1.4E6	1.7E6		Quinolactacin A: [M+Na]+: 0.494	0.494	C16H18N2O2	[M+Na]+		CCC(C)C1...	InChI=1S...	293.1260	2.5878	270.1368
271.1443	1.21	1.2E7	1.4E7		Quinolactacin A: [M+H]+: 0.838	0.838	C16H18N2O2	[M+H]+		CCC(C)C1...	InChI=1S...	271.1441	0.8980	270.1368
1255.6354	0.74	2.4E6	2.4E6		DACTINOMYCIN: [M+H]+: 0.937	0.937	C62H86N12O16	[M+H]+		Cc1c2oc3...	InChI=1S...	1255.6358	-0.3163	1254.6285
378.1917	0.74	1.0E6	1.1E6		7-(3,4-Dihydroxyphenyl)-1-(3-methoxy-4,...	0.678	C20H24O6	[M+NH4]+		COc1cc(C...	InChI=1S...	378.1911	1.6100	360.1573
484.1818	0.74	2.2E6	2.2E6		4-[(2S)-2-hydroxy-3-methyl-3-[[(2S,3R,4S...	0.794	C22H26O11	[M+NH4]+		CC(C)(OC...	InChI=1S...	484.1813	1.0313	466.1475
467.1554	0.74	4.3E6	4.8E6		4-[(2S)-2-hydroxy-3-methyl-3-[[(2S,3R,4S...	0.753	C22H26O11	[M+H]+		CC(C)(OC...	InChI=1S...	467.1548	1.2332	466.1475
265.1071	1.21	6.8E7	7.0E7		4,7,8-trimethoxy-3,5-dimethylchromen-2...	0.940	C14H16O5	[M+H]+		COc1c(O...	InChI=1S...	265.1071	0.3370	264.0998
287.1396	1.21	1.2E7	1.3E7											

Supplementary Fig. 5. Feature table in mzmine.
A screenshot of the mzmine feature table containing XIC shapes and compound annotations from the metadata sheet.

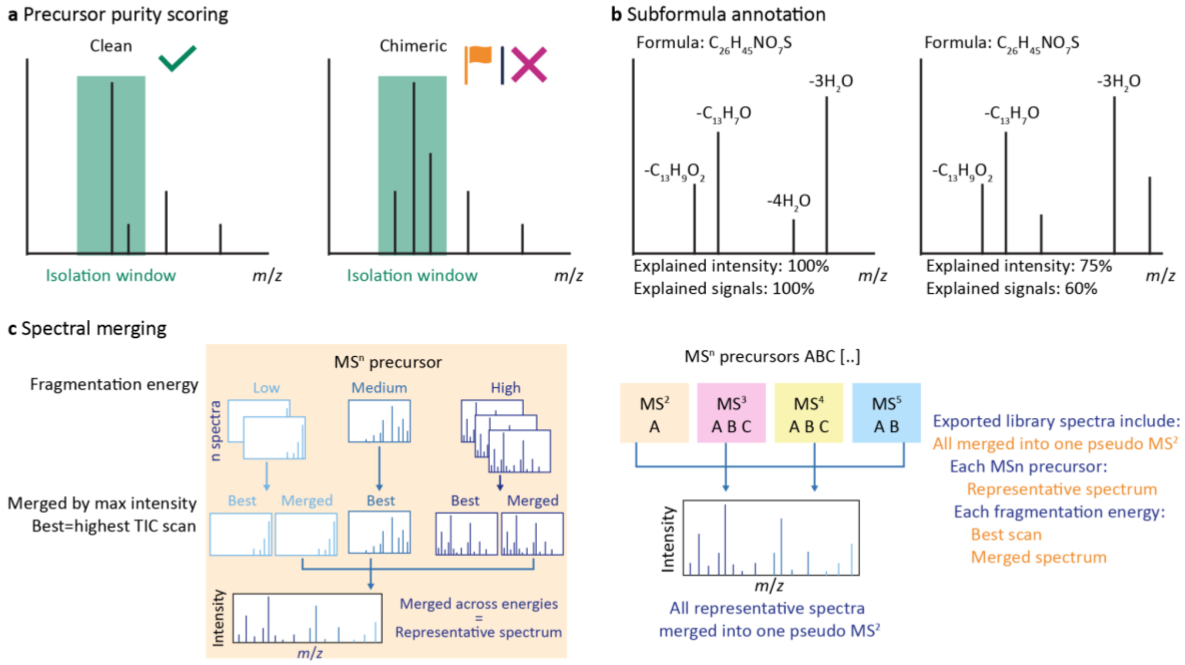

Supplementary Fig. 6. Quality check and merging within mzmine.
a, Purity score of the precursor by checking MS^1 data before and right after the tree acquisition within the isolation window. Chimeric spectra can be flagged or removed. **b**, Based on the given formula, fragmentation signals are annotated with a subformula (without taking the real structure into account). A threshold can be set to only export spectra, having an explained intensity or explained signals above a specific value. All scores are exported with each individual spectrum. **c**, Merging of MS^n spectra, including the same energy or various energies for one precursor ion, and the whole MS^n tree into a pseudo MS^2 spectrum.

Supplementary Note 5: MS^{n}Lib results

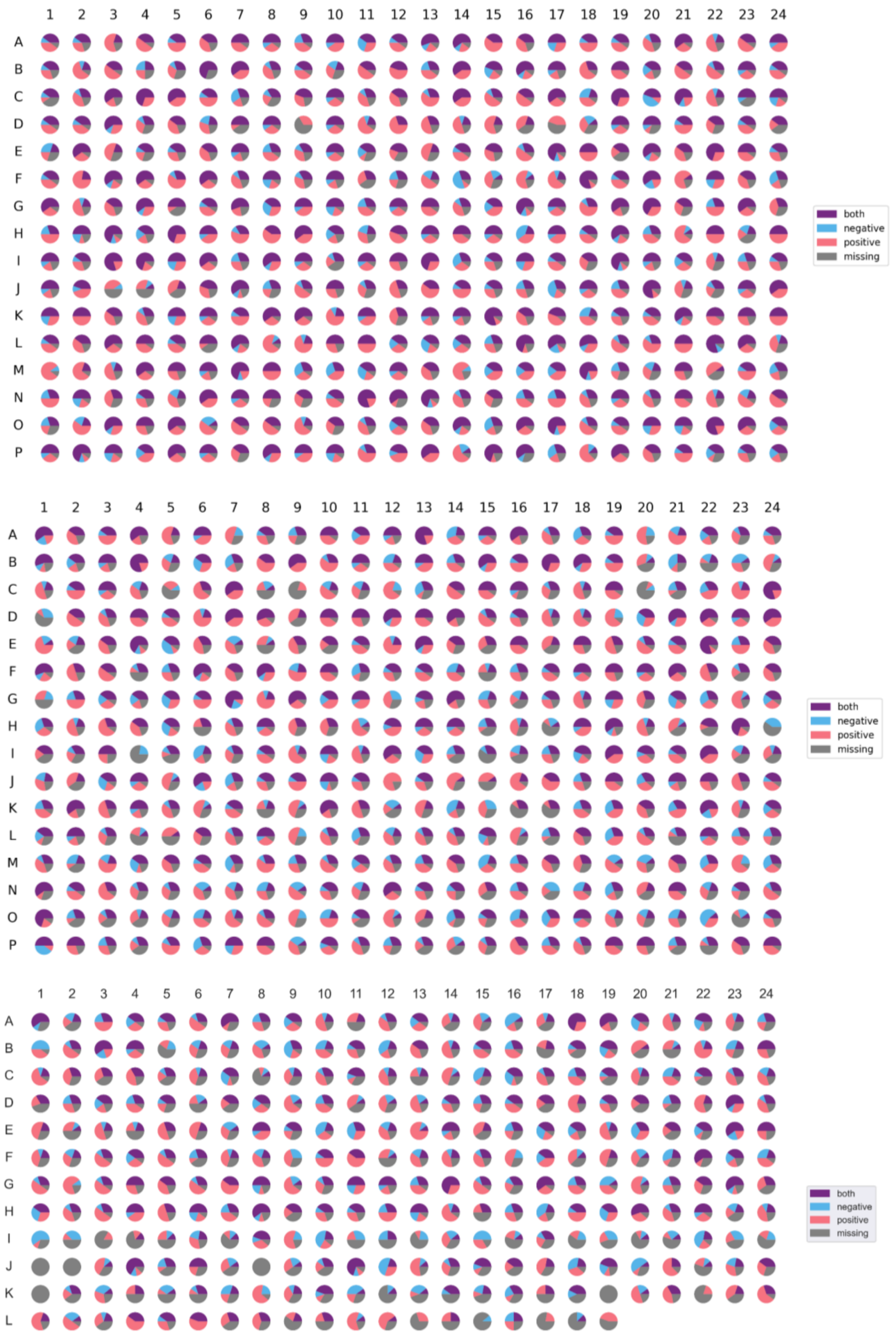

Supplementary Fig. 7. Ratios of detected compounds in both ion modes for 384-well plates 1-3 of the MCEBIO.

Each well contains a mixture of 10 compounds. Each compound was detected either in positive, negative, both ion modes, or remained missing during the analysis. The pie chart shows the detection ratio for all compounds in this well.

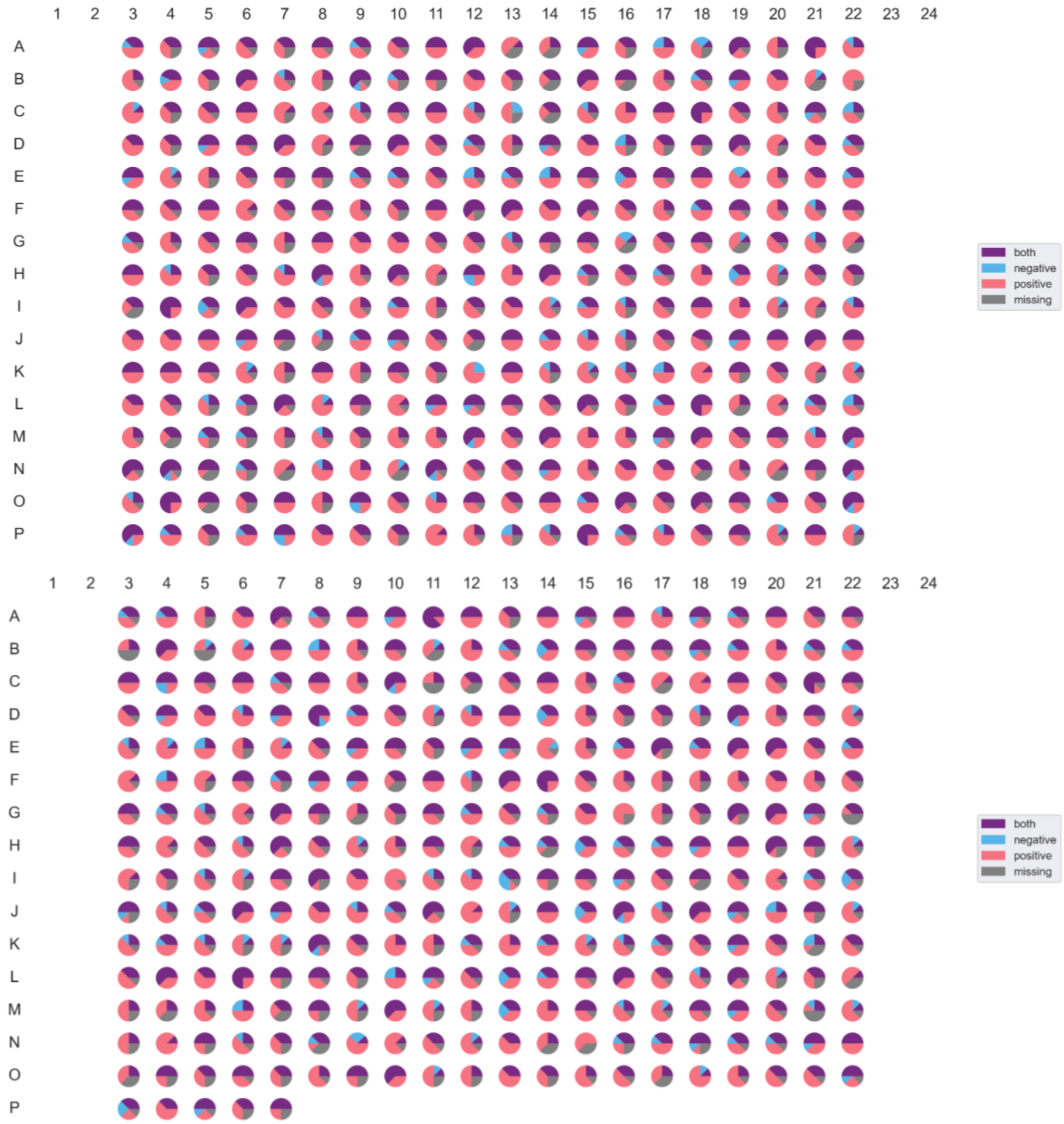

Supplementary Fig. 8. Ratios of detected compounds in both ion modes (both, only one, or missing) for 384-well plates 1-2 of the MCESCAF.

Each well contains a mixture of 8 compounds. Each compound was detected either in positive, negative, both ion modes, or remained missing during the analysis. The pie chart shows the detection ratio for all compounds in this well.

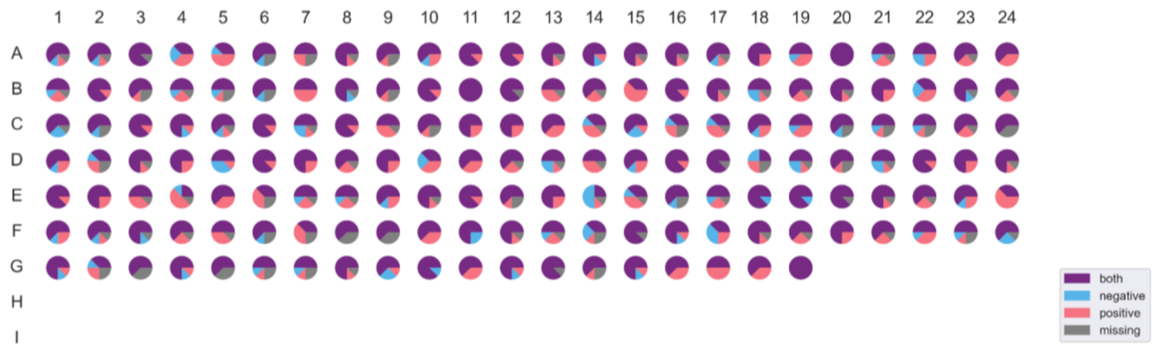

Supplementary Fig. 9. Ratios of detected compounds in both ion modes (both, only one, or missing) for 384-well plate 1 of the OTAVAPEP.

Each well contains a mixture of 8 compounds. Each compound was detected either in positive, negative, both ion modes, or remained missing during the analysis. The pie chart shows the detection ratio for all compounds in this well.

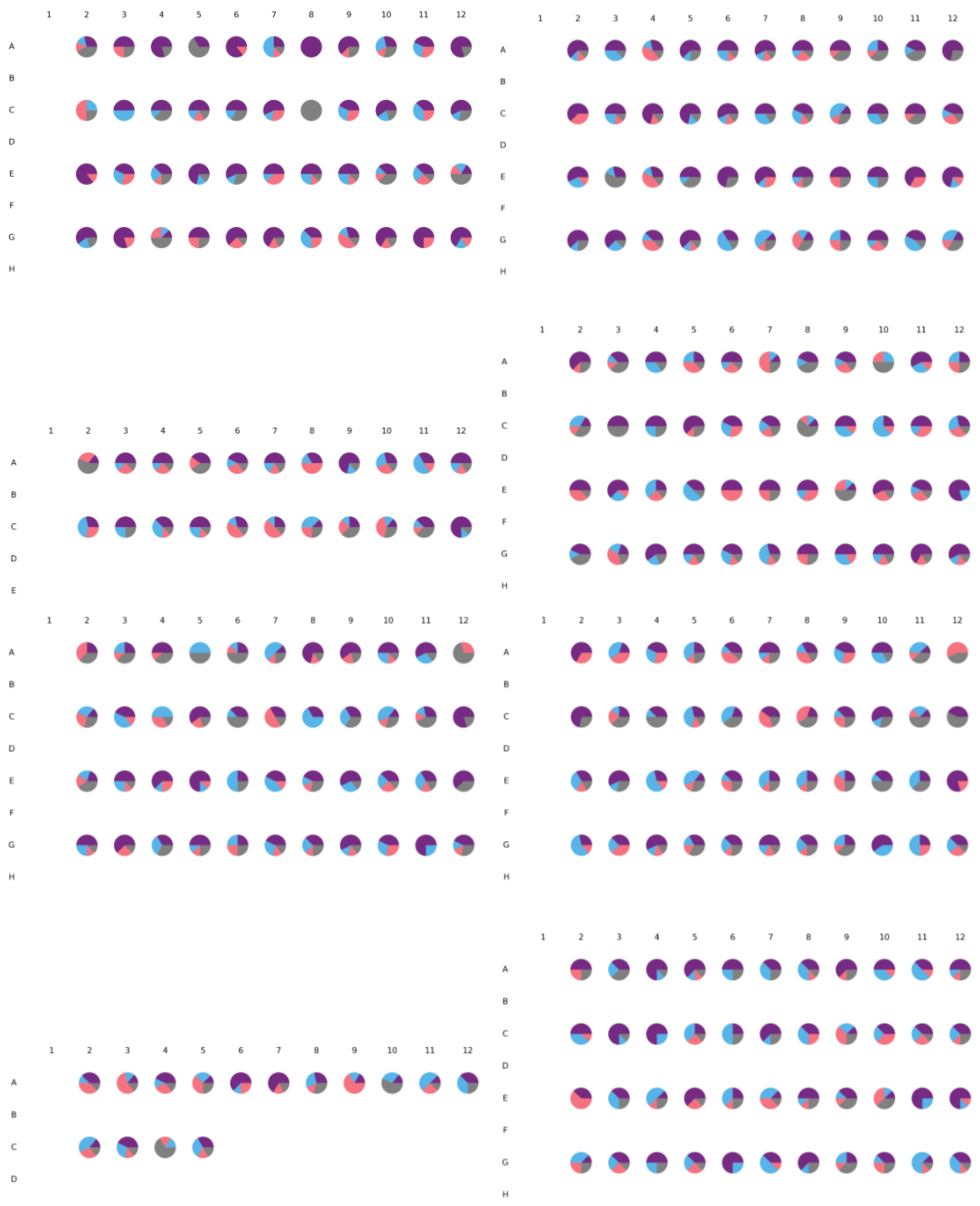

Supplementary Fig. 10. Ratios of detected compounds in both ion modes (both, only one, or missing) for 96-well plates 1-6 and 8-15 of the NIHNP.

Each well contains a mixture of up to 8 compounds. Each compound was detected either in positive, negative, both ion modes, or remained missing during the analysis. The pie chart shows the detection ratio for all compounds in this well.

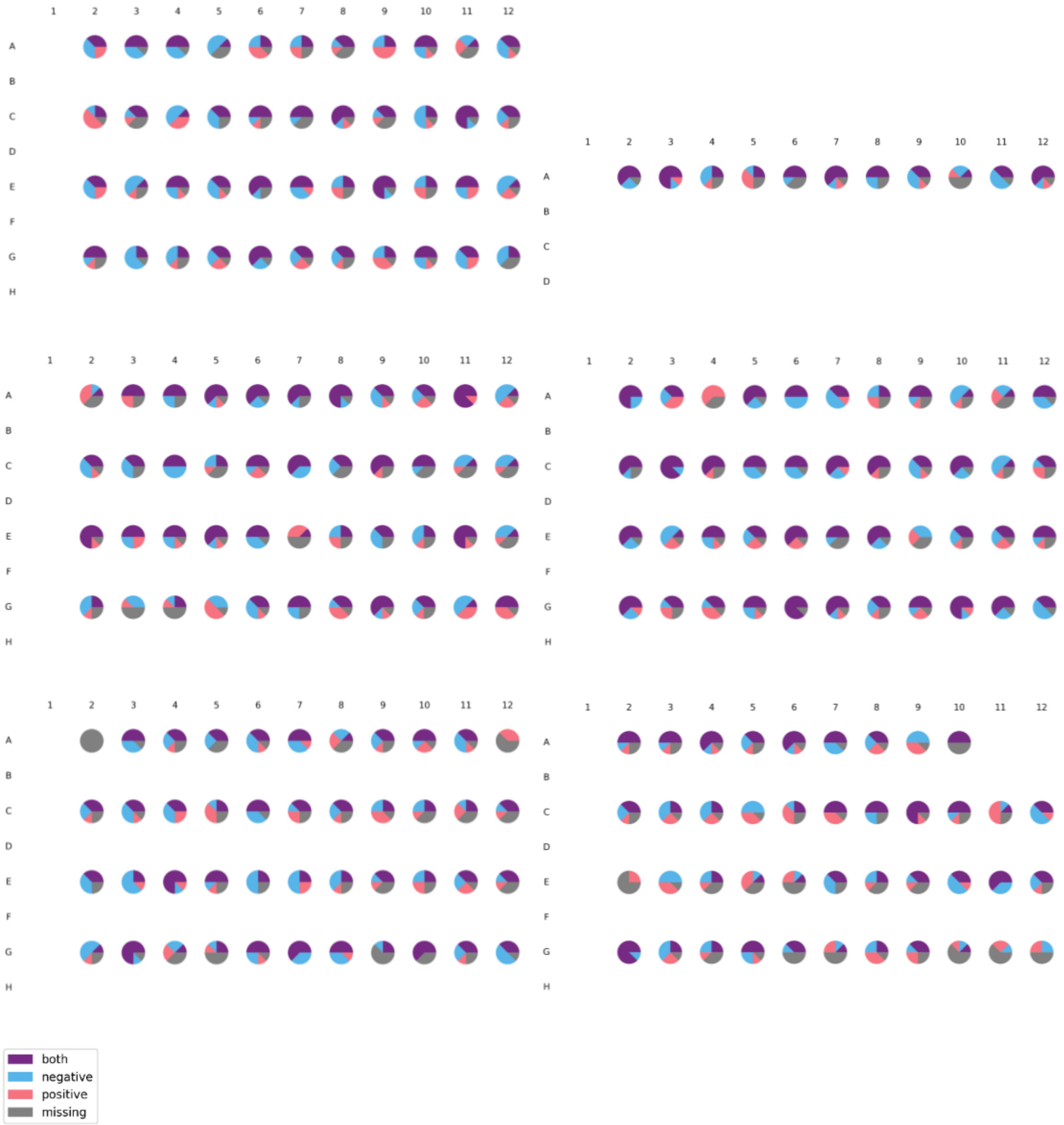

Supplementary Fig. 10 (continued). Ratios of detected compounds in both ion modes (both, only one, or missing) for 96-well plates 1-6 and 8-15 of the NIHNP.

Each well contains a mixture of up to 8 compounds. Each compound was detected either in positive, negative, both ion modes, or remained missing during the analysis. The pie chart shows the detection ratio for all compounds in this well.

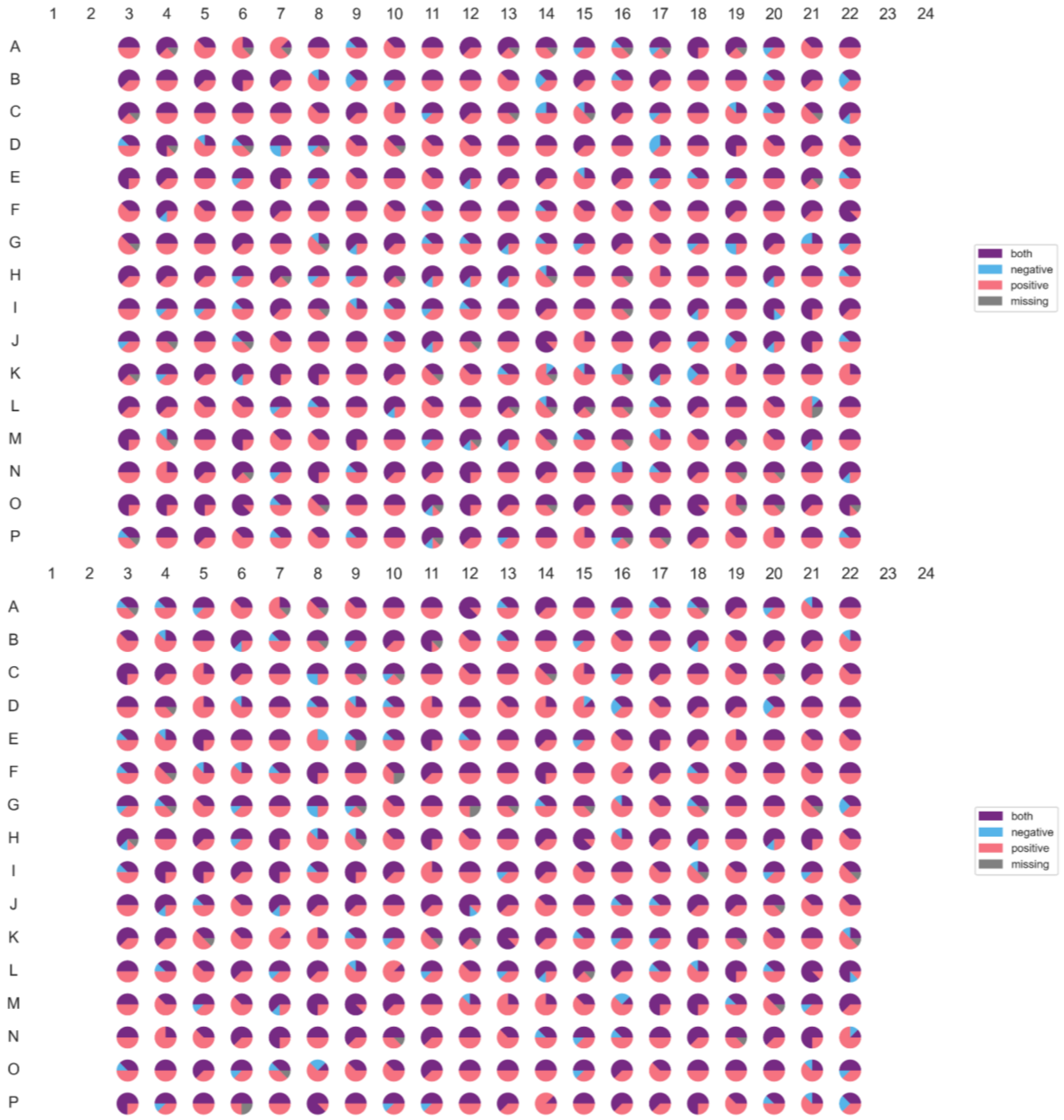

Supplementary Fig. 11. Ratios of detected compounds in both ion modes (both, only one, or missing) for 384-well plates 1-4 of the ENAMDISC.

Each well contains a mixture of up to 8 compounds. Each compound was detected either in positive, negative, both ion modes, or remained missing during the analysis. The pie chart shows the detection ratio for all compounds in this well.

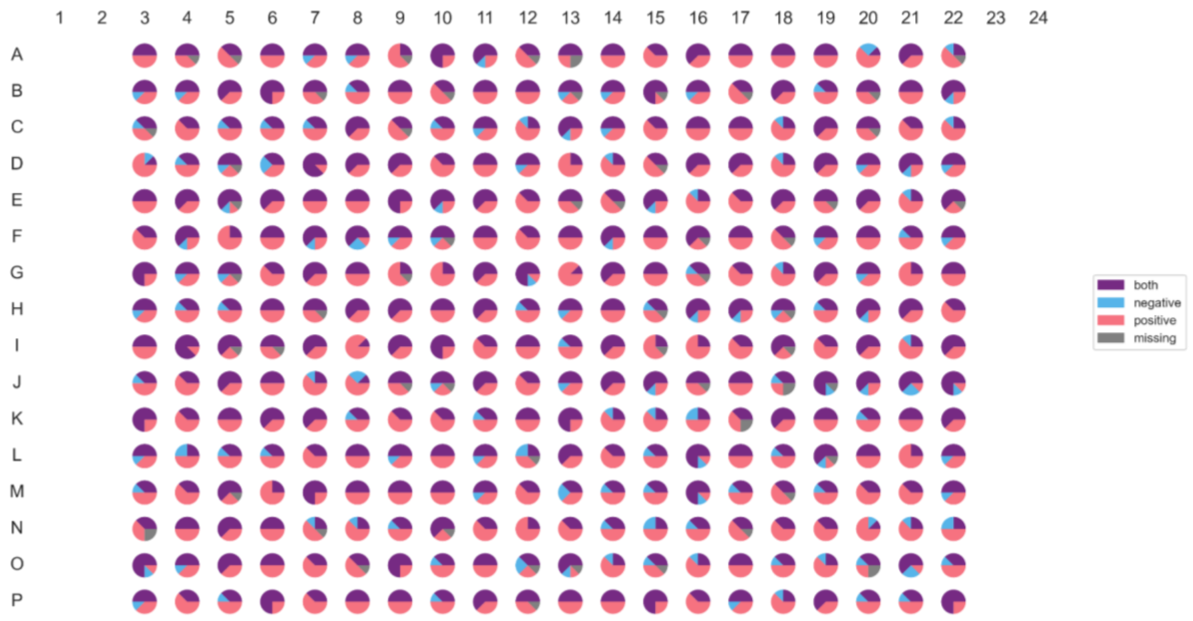

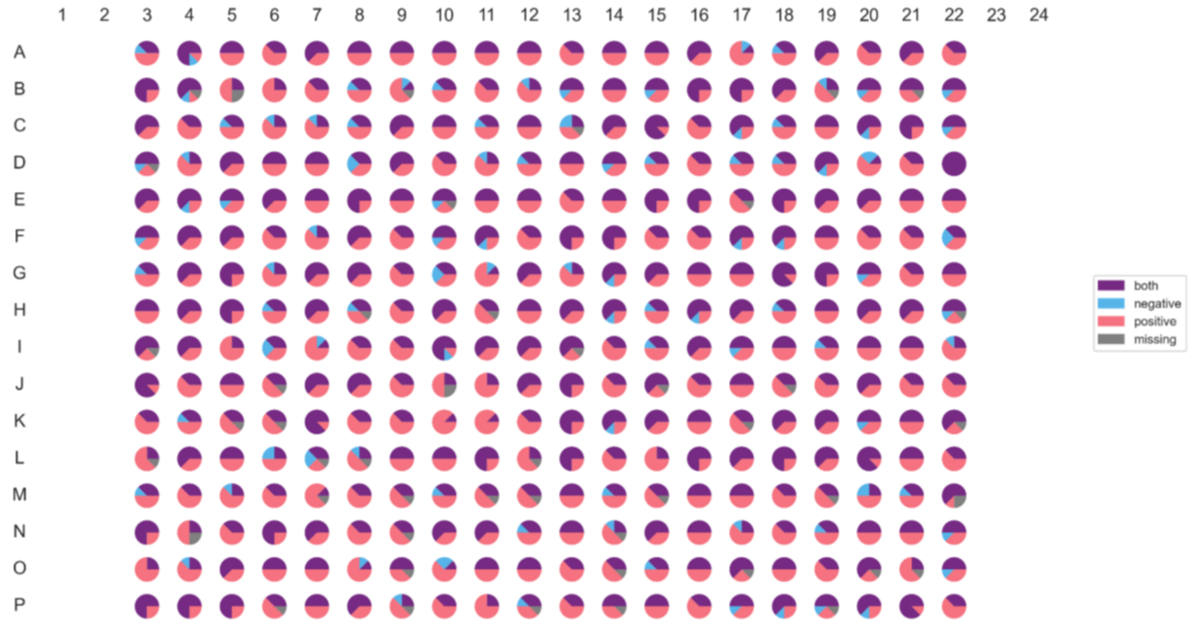

Supplementary Fig. 11 (continued). Ratios of detected compounds in both ion modes (both, only one, or missing) for 384-well plates 1-4 of the ENAMDISC.

Each well contains a mixture of up to 8 compounds. Each compound was detected either in positive, negative, both ion modes, or remained missing during the analysis. The pie chart shows the detection ratio for all compounds in this well.

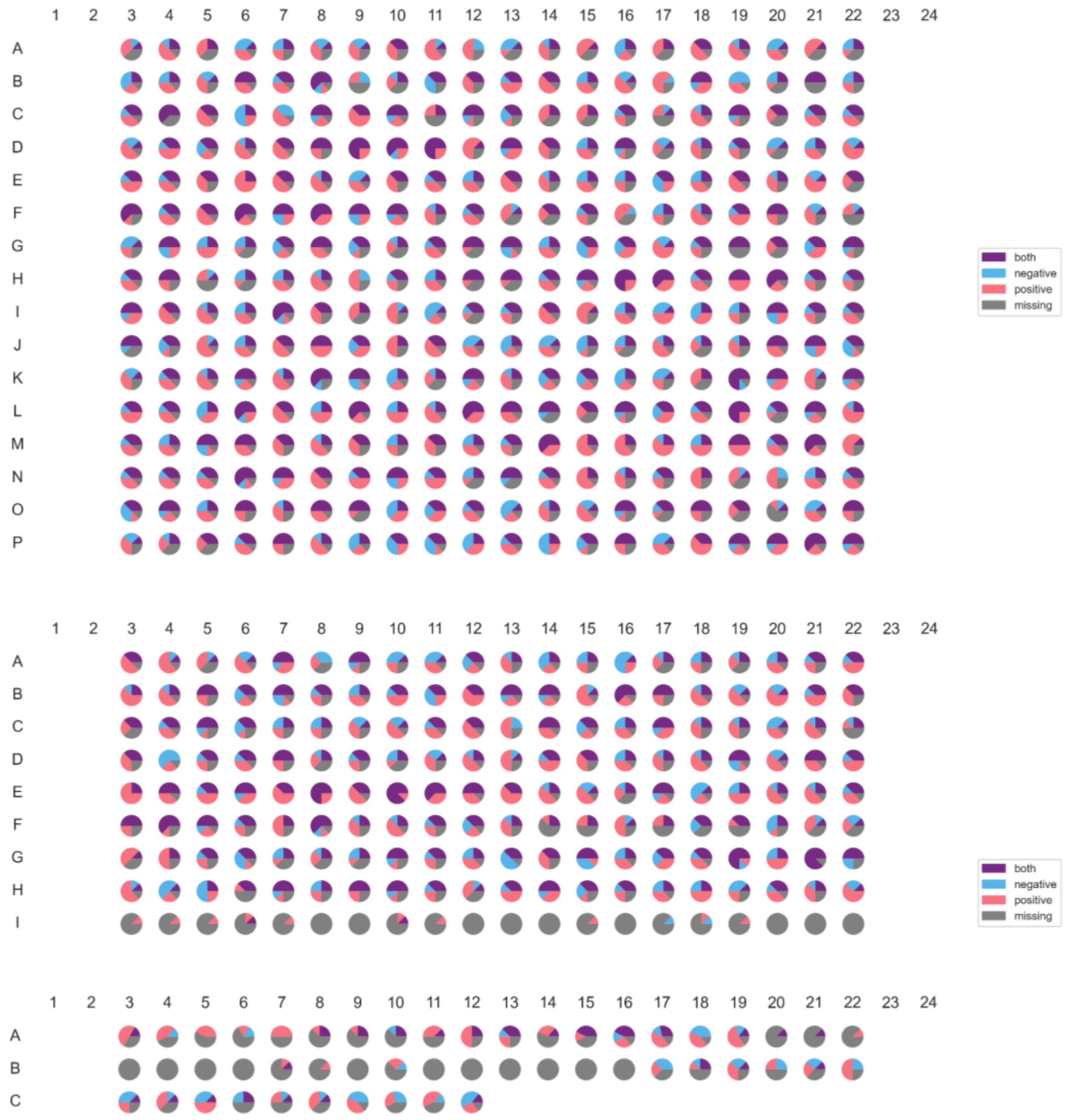

Supplementary Fig. 12. Ratios of detected compounds in both ion modes (both, only one, or missing) for 384-well plates 1-3 of the ENAMMOL.

Each well contains a mixture of up to 8 compounds. Each compound was detected either in positive, negative, both ion modes, or remained missing during the analysis. The pie chart shows the detection ratio for all compounds in this well.

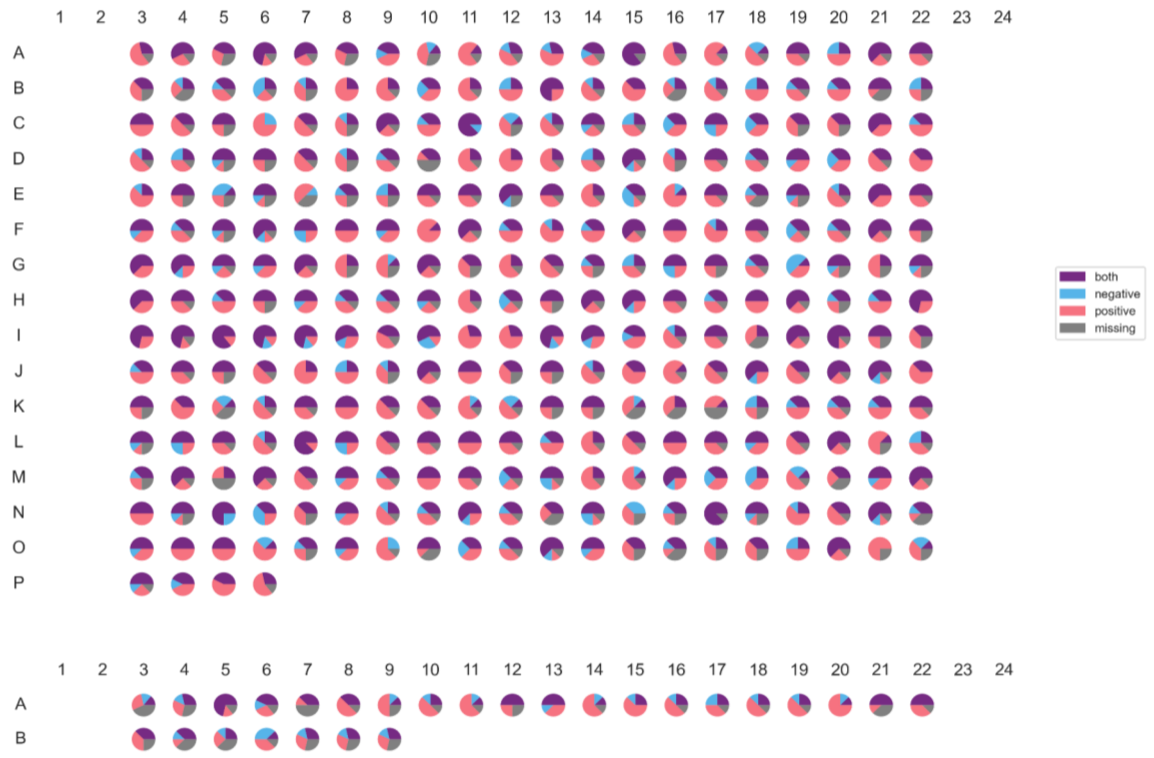

Supplementary Fig. 13. Ratios of detected compounds in both ion modes (both, only one, or missing) for 384-well plates 1-2 of the MCEDRUG.

Each well contains a mixture of up to 8 compounds. Each compound was detected either in positive, negative, both ion modes, or remained missing during the analysis. The pie chart shows the detection ratio for all compounds in this well.

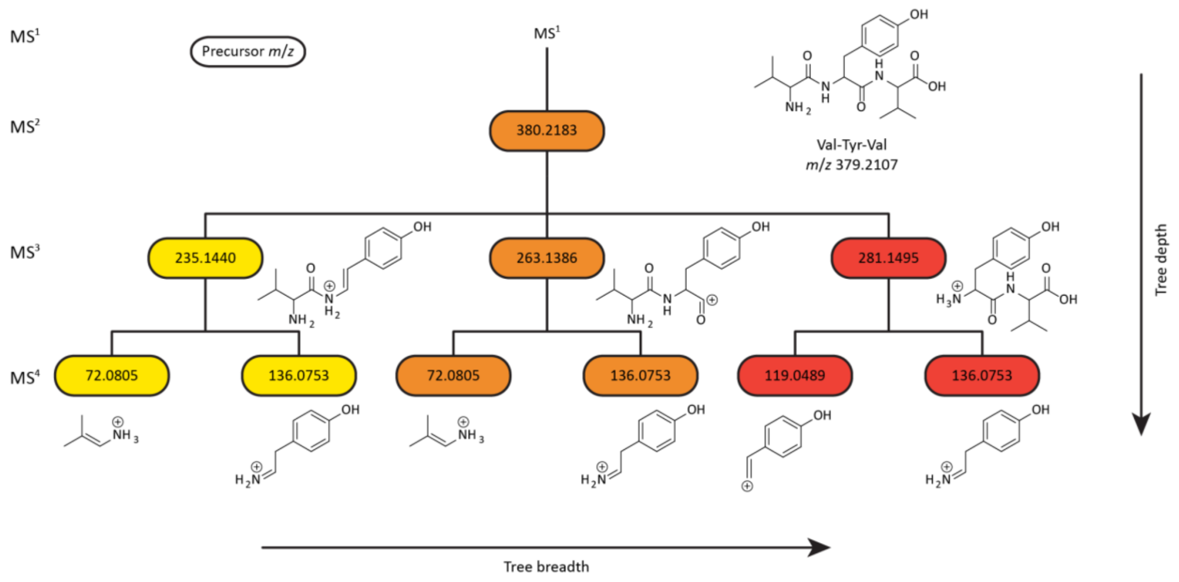

Supplementary Fig. 14. An example of an MS^n tree for the peptide Val-Tyr-Val.

Each mass is picked as a precursor for further fragmentation experiments (3 precursors for MS^3 and 2 precursors for MS^4).

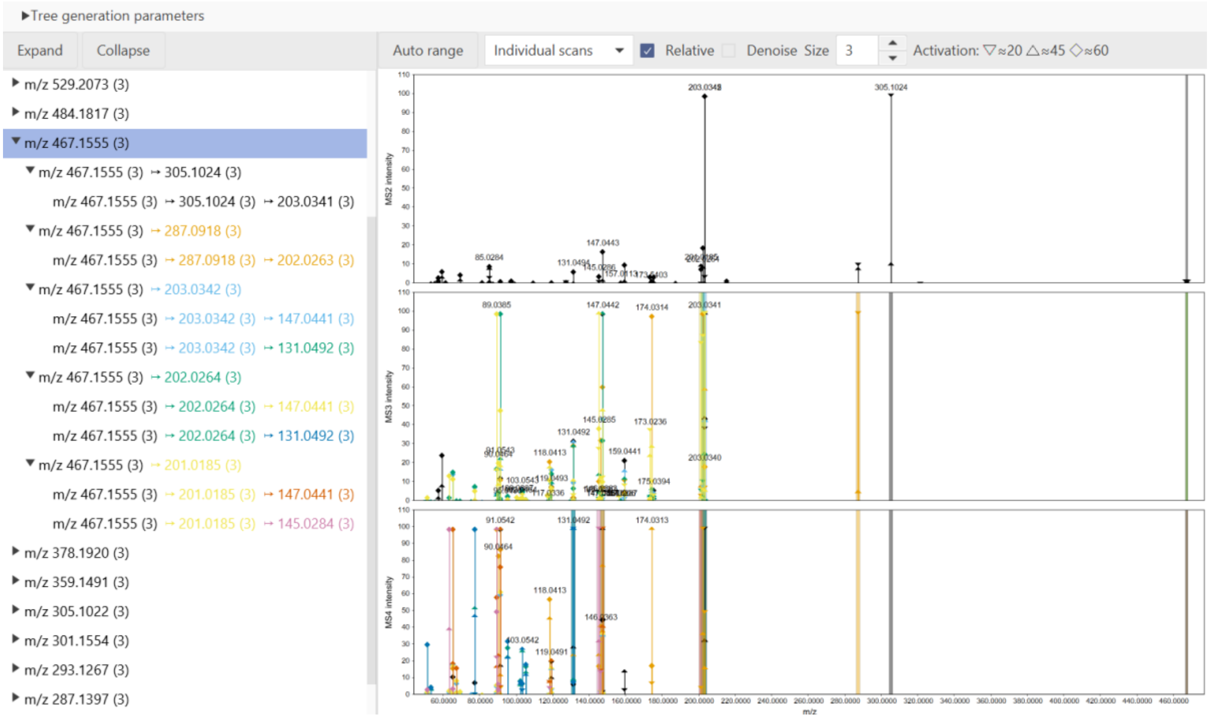

Supplementary Fig. 15. The MS^n tree visualizer in mzmine (screenshot).

The MS^n tree visualizer can be used to visualize multiple fragmentation spectra combined into one chart per MS level, here from MS levels 2, 3, and 4 from top to bottom. Different fragmentation energies are denoted by three different shapes depending on being closer to the lowest, median, or highest fragmentation energy found for this precursor ion. The colors correspond to the MS^n tree on the left on the same MS level.

Supplementary Note 6: Quality check for the automatic spectral extraction

We also tested the quality of the new spectral library with spectra originating from the MCEBIO library, acquired in positive ionization mode. In this evaluation, all 48,000 MS^2 spectra were matched against open spectral databases, and the chemical space only of the acquired spectra was mapped by feature-based molecular networking (FBMN)[16] using the GNPS web platform[17]. Here, we limited the matching to open libraries, enabling the same spectral matching parameters and workflow. The results are visualized in **Extended Data Fig. 5**. Here, each node represents one MS^2 spectrum from the MCEBIO positive data and the nodes are connected with an edge if the MS^2 spectra share a spectral similarity (cosine similarity ≥ 0.7 and at least four matched signals). Out of 48,000 spectra with multiple spectra acquired at different fragmentation energies per compound, only 20% showed a library match with a minimum cosine similarity of 0.6 and four matching fragment ions. This demonstrates the uniqueness of the acquired spectra and included compounds. To further evaluate the 20% matches, the same structure cleanup pipeline was used for the matched compounds (originating from the public spectral database) and we compared those structures with the actual structure. As a result, 68% showed a direct match based on the same InChIKey. As small structural changes result in different InChIKey strings, we added a comparison based on structural similarity assessed using the maximum common edge subgraph (MCES)[18] algorithm (https://github.com/AlBi-HHU/myopic-mces) and Tanimoto similarity. Briefly, MCES counts the number of structural modifications resulting in MCES 0 for identical structures and MCES 2 if the position of a functional group changes, as highlighted in **Extended Data Fig. 5b** for acetophenazine and its library match. The structure stored in the open domain has the acetyl group at another position on the ring, but the spectra remain similar, as expected. Most likely, however, the structure in the open spectral library was wrong, as the declared name was acetophenazine. In the case of Tanimoto similarity, 88% of all matches showed high similarity values of ≥ 0.85. For MCES, 94% of all matches were identical or highly similar with MCES < 4. With this information, almost all annotations based on spectral library matching correspond to the same or very similar structure, showing the correct export and public library matching of the newly created spectral data. On the other hand, 80% of our spectral library entries remained new compared to the open libraries spectra. It is important to note that the same compound may be in the public database, but differences in instruments, fragmentation energies, or settings can affect the library match. However, comparing the results of the compound coverage, around 74% of all acquired compounds are new compared to other open libraries (see **Fig. 2**), which is in good accordance with the number of unmatched spectra (see **Extended Data Fig. 5**).
We further evaluated the quality of the spectra based on the clustering of molecular families during FBMN (edges), where similar compound classes should fall into the same spectral subnetworks. This is the case for many clusters in the network and is highlighted in **Extended Data Fig. 5b** for the acetophenazine subnetwork, grouping compounds from the phenothiazine class. Here, acetophenazine connects two analog structures, namely piperacetazine and perphenazine, as they share the same core structure with only one modification. Both analogs remain unconnected, highlighting how modified cosine similarity primarily connects single modifications. Those networks demonstrate the spectral quality and correct exporting of the corresponding structures and names by the automatic library generation workflow. Finally, we emphasize that the libraries were acquired without chromatographic separation by flow injection-MS^n, essential for obtaining deep and wide MS^n

trees in a high throughput workflow. The compound libraries, sourced from various companies, were not individually tested for quality. Consequently, structural isomers, contaminants, and other isobaric interferences may be present during acquisition, annotation, and in the final MS^nLib. This challenge was already discussed in the LibGen article.[19]

Supplementary Note 7: Matching to a public metabolomics dataset

The newly generated MS^nLib was further evaluated by matching against a public metabolomics dataset (MSV000096589) in combination with the other open spectral libraries. This dataset analyzes bacterial community cultures incubated with various drugs. After mzmine data processing, the feature table contained 9078 features with 6913 including MS^2 experiments. The library matching parameters were set for higher confidence with at least 6 matching signals and a weighted cosine similarity of ≥0.85 to only include high-quality spectral matches. The other open libraries (MoNA, GNPS, MassBank EU) and MSnLib yielded 129 and 80 annotated features, respectively. The combined total number of annotations was 150 with 21 unique annotations by MS^nLib. The unique annotations were evaluated and compared against the list of 142 added drugs. Based on the first InChIKey block, 14 annotations correspond directly to the added drugs. The seven remaining matches may originate from microbial metabolism, e.g., ufiprazole as a metabolite of omeprazole, and were not further investigated. Library matching results and spectral mirror plots for these matches are collected in Supplementary File 10 and Supplementary File 11. These results on a real dataset further validate the quality of MS^nLib entries and the complementary nature to other open libraries.

Supplementary Note 8: Outlook

Looking ahead, we aim to acquire additional compound libraries and expand the covered chemical space by continuously adding to the free and open MSnLib resource. The primary challenge in constructing large-scale mass spectral libraries is access to compound libraries, which are often costly and required in small quantities. This necessitates collaboration. We are grateful to our collaborators, who have already provided 7 compound libraries, and are actively reaching out to community members to share commercial, purified, or synthesized compounds. MSnLib data has been acquired on a single Orbitrap ID-X instrument because of its MSn capabilities, however, we plan to build other MS2 libraries by including data from various instruments and fragmentation techniques, also capturing additional identifiers like retention time and ion mobility. This will enhance the diversity and utility of open mass spectral libraries. Substructure prediction by MassFrontier, CFM-ID,[20] or other tools may add additional value to the MS^nLib resource. However, substructure prediction requires extensive manual validation and we invite the mass spectrometry community to join our effort in developing solutions.

Importantly, we emphasize that library data can be dynamically regenerated with different spectral processing parameters and filters to enable precise control of the generated library entries. This capability is expected to improve model training and enable the customization of libraries for specific machine learning models. All necessary information for regenerating the MS^n library, namely the data, compound metadata, and mzmine workflow configuration, is openly shared. This is a paradigm shift in the generation of spectral libraries, leading to greater traceability of the library entries to raw data using the Universal Spectrum Identifier (USI) with transparent processing parameters. A recent study validated our library quality by checking compound and spectral metadata in all public spectral libraries. These checks removed over 31,000 spectra each from GNPS, MoNA and MassBankEU, while MS^nLib failed checks for 58 entries.[21] This shows that automatically curated libraries may improve the overall quality compared to historically grown libraries that relied on manual curation by contributors and often lost links to the processing history and original raw data.

We expect that the demonstrated high-throughput method will facilitate rapid growth in the public availability of reference spectra and that this unique resource will stimulate the development of new machine learning-based tools leveraging the spectral tree dependency of fragment ions across multi-stage fragmentation. Newer models for MS2DeepScore[22] and MS2Query[23] are already trained with the new MS^nLib (MS^2 only) together with GNPS data. The DreaMS project uses MS^nLib for evaluation and fine-tuning of their model for specialized tasks, such as predicting fluorinated compounds.[24]

References

1. Rutz, A. *et al.* The LOTUS initiative for open knowledge management in natural products research. *Elife* **11**, (2022).
2. Bento, A. P. *et al.* An open source chemical structure curation pipeline using RDKit. *J. Cheminform.* **12**, 51 (2020).
3. Kim, S. *et al.* PubChem 2023 update. *Nucleic Acids Res.* **51**, D1373–D1380 (2023).
4. Davies, M. *et al.* ChEMBL web services: streamlining access to drug discovery data and utilities. *Nucleic Acids Res.* **43**, W612–20 (2015).
5. Zdrazil, B. *et al.* The ChEMBL Database in 2023: a drug discovery platform spanning multiple bioactivity data types and time periods. *Nucleic Acids Res.* **52**, D1180–D1192 (2024).
6. Chambers, J. *et al.* UniChem: a unified chemical structure cross-referencing and identifier tracking system. *J. Cheminform.* **5**, 3 (2013).
7. Wishart, D. S. *et al.* DrugBank: a comprehensive resource for in silico drug discovery and exploration. *Nucleic Acids Res.* **34**, D668–72 (2006).
8. Ursu, O. *et al.* DrugCentral: online drug compendium. *Nucleic Acids Res.* **45**, D932–D939 (2017).
9. Corsello, S. M. *et al.* The Drug Repurposing Hub: a next-generation drug library and information resource. *Nat. Med.* **23**, 405–408 (2017).
10. van Santen, J. A. *et al.* The Natural Products Atlas 2.0: a database of microbially-derived natural products. *Nucleic Acids Res.* **50**, D1317–D1323 (2022).
11. Kim, H. W. *et al.* NPClassifier: A Deep Neural Network-Based Structural Classification Tool for Natural Products. *J. Nat. Prod.* **84**, 2795–2807 (2021).
12. Djoumbou Feunang, Y. *et al.* ClassyFire: automated chemical classification with a comprehensive, computable taxonomy. *J. Cheminform.* **8**, 61 (2016).
13. Dührkop, K. *et al.* SIRIUS 4: a rapid tool for turning tandem mass spectra into metabolite structure information. *Nat. Methods* **16**, 299–302 (2019).
14. Schmid, R. *et al.* Ion identity molecular networking for mass spectrometry-based metabolomics in the GNPS environment. *Nat. Commun.* **12**, 3832 (2021).
15. Deutsch, E. W. *et al.* Universal Spectrum Identifier for mass spectra. *Nat. Methods* **18**, 768–770 (2021).
16. Nothias, L.-F. *et al.* Feature-based molecular networking in the GNPS analysis environment. *Nat. Methods* **17**, 905–908 (2020).
17. Wang, M. *et al.* Sharing and community curation of mass spectrometry data with Global Natural Products Social Molecular Networking. *Nat. Biotechnol.* **34**, 828–837 (2016).
18. Kretschmer, F., Seipp, J., Ludwig, M., Klau, G. W. & Böcker, S. Coverage bias in small molecule machine learning. *Nat. Commun.* **16**, (2025).
19. Kong, F., Keshet, U., Shen, T., Rodriguez, E. & Fiehn, O. LibGen: Generating high quality spectral libraries of natural products for EAD-, UVPD-, and HCD-high resolution mass spectrometers. *Anal. Chem.* **95**, 16810–16818 (2023).
20. Wang, F. *et al.* CFM-ID 4.0: More Accurate ESI-MS/MS Spectral Prediction and Compound Identification. *Anal. Chem.* **93**, 11692–11700 (2021).
21. de Jonge, N. F. *et al.* Reproducible MS/MS library cleaning pipeline in matchms. *J. Cheminform.* **16**, 88 (2024).
22. Huber, F., van der Burg, S., van der Hooft, J. J. J. & Ridder, L. MS2DeepScore: a novel

deep learning similarity measure to compare tandem mass spectra. *J. Cheminform.* **13**, 84 (2021).
23. de Jonge, N. F. *et al.* MS2Query: reliable and scalable MS2 mass spectra-based analogue search. *Nat. Commun.* **14**, 1752 (2023).
24. Bushuiev, R., Bushuiev, A., Samusevich, R., Šivic, J. & Pluskal, T. Emergence of molecular structures from self-supervised learning on mass spectra. *ChemRxiv* (2023) doi:10.26434/chemrxiv-2023-kss3r.
