# Supplementary material for: MSnLib: efficient generation of open multi-stage fragmentation mass spectral libraries
Source: Nat Methods. 2025 Sep 15;22(10):2028–31. doi: 10.1038/s41592-025-02813-0 (PMC12510872; doi:10.1038/s41592-025-02813-0)
Supplement: Supplementary file 2 — Reporting Summary [file 41592_2025_2813_MOESM2_ESM.pdf]

nature portfolio

Corresponding author(s): Robin Schmid, Tomáš Pluskal

Last updated by author(s): Apr 24, 2025

Reporting Summary

Nature Portfolio wishes to improve the reproducibility of the work that we publish. This form provides structure for consistency and transparency in reporting. For further information on Nature Portfolio policies, see our Editorial Policies and the Editorial Policy Checklist.

Statistics

For all statistical analyses, confirm that the following items are present in the figure legend, table legend, main text, or Methods section.

n/a	Confirmed	
☒	☐	The exact sample size (*n*) for each experimental group/condition, given as a discrete number and unit of measurement
☒	☐	A statement on whether measurements were taken from distinct samples or whether the same sample was measured repeatedly
☒	☐	The statistical test(s) used AND whether they are one- or two-sided<br>*Only common tests should be described solely by name; describe more complex techniques in the Methods section.*
☒	☐	A description of all covariates tested
☒	☐	A description of any assumptions or corrections, such as tests of normality and adjustment for multiple comparisons
☒	☐	A full description of the statistical parameters including central tendency (e.g. means) or other basic estimates (e.g. regression coefficient) AND variation (e.g. standard deviation) or associated estimates of uncertainty (e.g. confidence intervals)
☒	☐	For null hypothesis testing, the test statistic (e.g. *F*, *t*, *r*) with confidence intervals, effect sizes, degrees of freedom and *P* value noted<br>*Give P values as exact values whenever suitable.*
☒	☐	For Bayesian analysis, information on the choice of priors and Markov chain Monte Carlo settings
☒	☐	For hierarchical and complex designs, identification of the appropriate level for tests and full reporting of outcomes
☒	☐	Estimates of effect sizes (e.g. Cohen's *d*, Pearson's *r*), indicating how they were calculated

Our web collection on statistics for biologists contains articles on many of the points above.

Software and code

Policy information about availability of computer code

Data collection	Xcalibur 4.5.445.18
Data analysis	MZmine 4.0.8, https://github.com/mzmine/mzmine3, https://github.com/mzmine/mzmine_documentation, Python 3.10, https://github.com/corinnabrungs/msn_tree_library, https://github.com/corinnabrungs/msn_tree_library/tree/master/notebooks, https://gnps.ucsd.edu/ProteoSAFe/status.jsp?task=c05d34fb31ab4ee99293e722fb7eb83d

For manuscripts utilizing custom algorithms or software that are central to the research but not yet described in published literature, software must be made available to editors and reviewers. We strongly encourage code deposition in a community repository (e.g. GitHub). See the Nature Portfolio guidelines for submitting code & software for further information.

Data

Policy information about availability of data

All manuscripts must include a data availability statement. This statement should provide the following information, where applicable:

- Accession codes, unique identifiers, or web links for publicly available datasets
- A description of any restrictions on data availability
- For clinical datasets or third party data, please ensure that the statement adheres to our policy

All metadata files, one per compound library, and mzmine batch files for library processing were uploaded to the MERLIN (Mass spEctRal LIbrary Network) GitHub repository (https://github.com/merlin-ms) under the MIT license. All acquired flow injection–Orbitrap MSn files were deposited as .mzML and .raw files in the

Zenodo datasets: 10966280 (.mzML positive and negative), 10966404 (.raw positive), and 10967081 (.raw negative) under the CC BY 4.0 license, as well as to MassIVE MSV000094528 under the CC0 1.0 license. The mass spectral libraries included in MSnLib were deposited as .mgf and .json in the Zenodo dataset 11163380 under the CC BY 4.0 license. Here, each spectral library for the individual compound libraries is uploaded as MS2 only or the full MSn library. The MS2 libraries contain the best and merged spectra for all acquired MS2 spectra, including the pseudo-MS2, where the whole fragmentation tree is combined into a single spectrum. Polarities are kept separated, resulting in 4 entries for each compound library and library format. Additionally, the MS2 data of MCEBIO, MCESCAF, NIHNP, and OTAVPEP are uploaded as a reference library in GNPS (https://external.gnps2.org/gnpslibrary) in the form of a default gold-level library named MSNLIB-POSITIVE and MSNLIB-NEGATIVE. We recommend using the Zenodo libraries as they contain more metadata and link back to the original data by Universal Spectrum identifier (USI).
DrugBank lookup is an optional step, and the data is accessible on request. More information is available on the project website (https://go.drugbank.com).
DrugCentral lookup is an optional step, and their whole database can be downloaded as a PostgreSQL dump from the company's website (https://drugcentral.org).
LOTUS lookup is an optional step, and the whole LOTUS dataset is incorporated into WIKIDATA. The metadata cleanup script contains a prefect flow to download all relationships from WIKIDATA. Simply run the prepare_wikidata_lotus_data_prefect.py script (https://github.com/corinnabrungs/msn_tree_library).
Broad Institute lookup is an optional step. The drug information can be downloaded as a .txt file from the institute's website (https://repo-hub.broadinstitute.org/repurposing).
The FBMN result can be accessed on GNPS:
https://gnps.ucsd.edu/ProteoSAFe/status.jsp?task=c05d34fb31ab4ee99293e722fb7eb83d
Experimental public libraries were downloaded from the corresponding webpages:
GNPS = ALL_GNPS_NO_PROPAGATED (https://external.gnps2.org/gnpslibrary, access 08.12.2023)
MassBankEU = MASSBANK_NIST
(https://github.com/MassBank/MassBank-data/releases/tag/2023.11, access 08.12.2023)
MassBank North America = LC-MS/MS Spectra
(https://mona.fiehnlab.ucdavis.edu/downloads, access 08.12.2023)
The dataset used for the library evaluation can be found in MassIVE under MSV000096589. Here, we only used a subset of group 1 (group1_B*.mzML) for the analysis. The mzmine batch file is supplied as Supplementary File 9 and results of this evaluation as Supplementary File 10 and 11.

Human research participants

Policy information about studies involving human research participants and Sex and Gender in Research.

Reporting on sex and gender	N/A
Population characteristics	N/A
Recruitment	N/A
Ethics oversight	N/A

Note that full information on the approval of the study protocol must also be provided in the manuscript.

Field-specific reporting

Please select the one below that is the best fit for your research. If you are not sure, read the appropriate sections before making your selection.

☒ Life sciences ☐ Behavioural & social sciences ☐ Ecological, evolutionary & environmental sciences

For a reference copy of the document with all sections, see nature.com/documents/nr-reporting-summary-flat.pdf

Life sciences study design

All studies must disclose on these points even when the disclosure is negative.

Sample size	9060 injections for 37,829 reference standards
Data exclusions	No
Replication	No
Randomization	No
Blinding	No

Reporting for specific materials, systems and methods

We require information from authors about some types of materials, experimental systems and methods used in many studies. Here, indicate whether each material, system or method listed is relevant to your study. If you are not sure if a list item applies to your research, read the appropriate section before selecting a response.

Materials & experimental systems

n/a	Involved in the study
☒	☐ Antibodies
☒	☐ Eukaryotic cell lines
☒	☐ Palaeontology and archaeology
☒	☐ Animals and other organisms
☒	☐ Clinical data
☒	☐ Dual use research of concern

Methods

n/a	Involved in the study
☒	☐ ChIP-seq
☒	☐ Flow cytometry
☒	☐ MRI-based neuroimaging
