# Supplementary figures and images for: MSnLib: efficient generation of open multi-stage fragmentation mass spectral libraries
Source: Nat Methods. 2025 Sep 15;22(10):2028–31. doi: 10.1038/s41592-025-02813-0 (PMC12510872; doi:10.1038/s41592-025-02813-0)

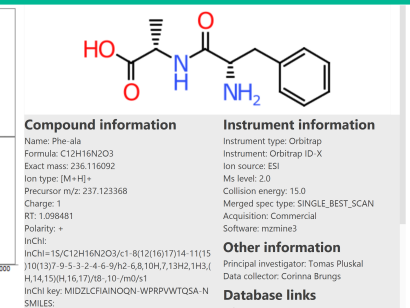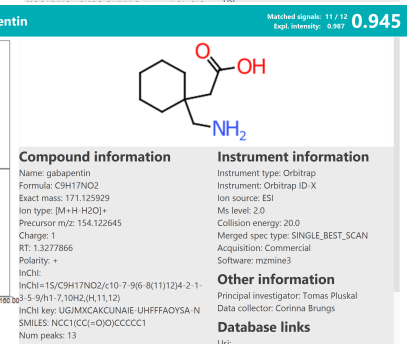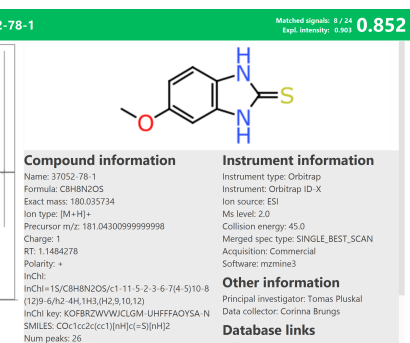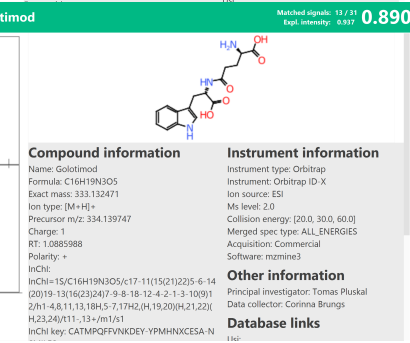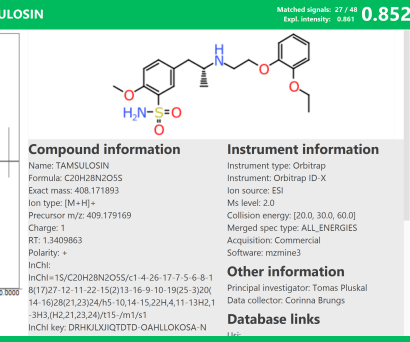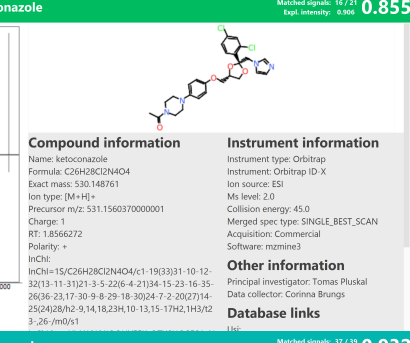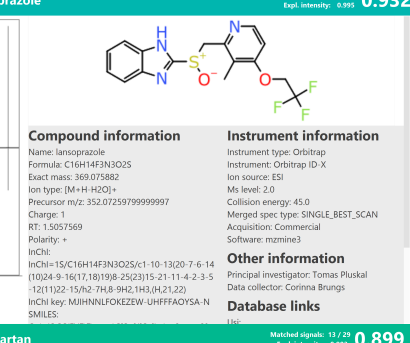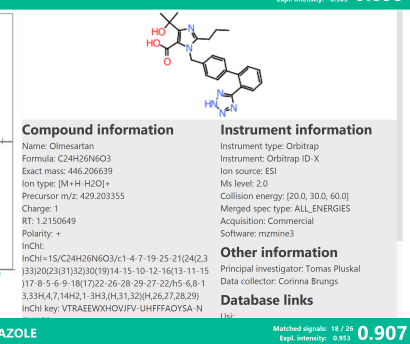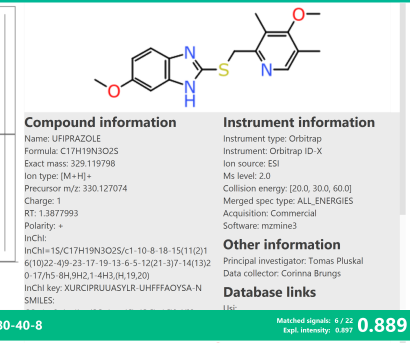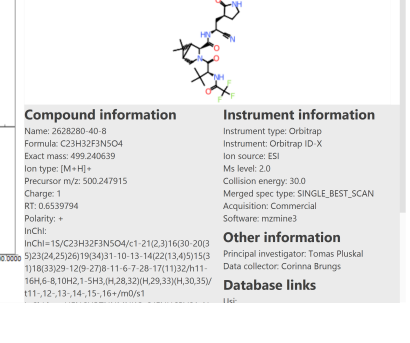

Supplement: Supplementary file 14 — Results of the annotation of MassIVE dataset MSV000096589 – mirror plots [file 41592_2025_2813_MOESM14_ESM.pdf]
